# Impact of Acute Sleep Deprivation on Sarcasm Detection

**DOI:** 10.1371/journal.pone.0140527

**Published:** 2015-11-04

**Authors:** Gaétane Deliens, Fanny Stercq, Alison Mary, Hichem Slama, Axel Cleeremans, Philippe Peigneux, Mikhail Kissine

**Affiliations:** 1 CO3—Consciousness, Cognition & Computation at CRCN—Center for Research in Cognition and Neurosciences and UNI—ULB Neurosciences Institute, Université libre de Bruxelles (ULB), Brussels, Belgium; 2 UR2NF—Neuropsychology and Functional Neuroimaging Research Group at CRCN—Center for Research in Cognition and Neurosciences and UNI—ULB Neurosciences Institute, Université libre de Bruxelles (ULB), Brussels, Belgium; 3 ACTE—Autism in Context: Theory and Experience at LaDisco—Center of Research in Linguistics, Université libre de Bruxelles (ULB), Brussels, Belgium; Institutes for Behavior Resources and Johns Hopkins University School of Medicine, UNITED STATES

## Abstract

There is growing evidence that sleep plays a pivotal role on health, cognition and emotional regulation. However, the interplay between sleep and social cognition remains an uncharted research area. In particular, little is known about the impact of sleep deprivation on sarcasm detection, an ability which, once altered, may hamper everyday social interactions. The aim of this study is to determine whether sleep-deprived participants are as able as sleep-rested participants to adopt another perspective in gauging sarcastic statements. At 9am, after a whole night of sleep (n = 15) or a sleep deprivation night (n = 15), participants had to read the description of an event happening to a group of friends. An ambiguous voicemail message left by one of the friends on another's phone was then presented, and participants had to decide whether the recipient would perceive the message as sincere or as sarcastic. Messages were uttered with a neutral intonation and were either: (1) sarcastic from both the participant’s and the addressee’s perspectives (i.e. both had access to the relevant background knowledge to gauge the message as sarcastic), (2) sarcastic from the participant’s but not from the addressee’s perspective (i.e. the addressee lacked context knowledge to detect sarcasm) or (3) sincere. A fourth category consisted in messages sarcastic from both the participant’s and from the addressee’s perspective, uttered with a sarcastic tone. Although sleep-deprived participants were as accurate as sleep-rested participants in interpreting the voice message, they were also slower. Blunted reaction time was not fully explained by generalized cognitive slowing after sleep deprivation; rather, it could reflect a compensatory mechanism supporting normative accuracy level in sarcasm understanding. Introducing prosodic cues compensated for increased processing difficulties in sarcasm detection after sleep deprivation. Our findings support the hypothesis that sleep deprivation might damage the flow of social interactions by slowing perspective-taking processes.

## Introduction

There is a pervasive body of evidence that sleep debt—a common issue in modern society [1]—exerts detrimental effects on health [2,3], cognitive functioning [4] and emotional regulation [5]. It is plausible that such effects lead, in turn, to alterations in interpersonal functioning and social interactions. However, little is known about the effects of sleep loss on the pragmatic, socially relevant, aspects of language interpretation. In the present study, we focused on the impact of sleep deprivation on sarcasm detection, an uncharted area of inquiry to the best of our knowledge. Sarcasm is a common pragmatic phenomenon serving many complex social functions. For instance, use of sarcasm makes it possible to convey a criticism indirectly, softening the acerbity of the criticism and promoting a relaxed atmosphere with a touch of humor (see [6]). Accordingly, in order to respond appropriately during social interactions it is often crucial to understand whether the speaker’s message should be perceived as sincere or sarcastic [7,8].

Among the few studies focusing on the effects of sleep on social cognition, Butt, Ouarda, Quan, Pentland, and Khayal [9] used Bluetooth proximity sensing over mobile phones to analyze the relationship between sleep parameters and social exposure. Their results indicate that sleep patterns modulate the following day's sociability, as social interactions were shorter in duration after a night with a lower percentage of Slow Wave Sleep (SWS) and Rapid-Eye Movement (REM) Sleep. Since physical appearance motivates social interactions (for a meta-analysis see [10]), one possible explanation for this reduction in social exposure is the deleterious effect of sleep deprivation on facial attractiveness [11]. An alternative explanation, however, is that sleep debt alters the *quality* of social interactions, eventually leading to shortened social exchanges. Further indication that sleep deprivation may impact the quality of social functioning comes from the fact that individuals with social-emotional functioning difficulties, such as autism spectrum disorder, alexithymia or schizophrenia, frequently encounter co-occurring sleep difficulties [12,13,14]. Likewise, decreased restorative sleep has been observed in individuals who reported higher feelings of loneliness [15].

Various factors may be responsible for this decrease in the quality of social interactions observed in people lacking sleep, such as the well-documented worsening of mood after sleep deprivation [16,17,18,19], the exacerbation of negative emotions in response to unpleasant events, the lower rate of positive responses to pleasant events [20], and an increased impulsive/aggressive behavior in response to frustrating situations [21]. However, lack of sleep may not only alter affective reactivity but also exert a deleterious impact on the perception of other people’s minds—a skill crucial for successful social interactions. Decreased speed and accuracy were evidenced in the recognition of facial emotions after a night of total sleep deprivation [22,23]. Accordingly, sleep-deprived participants report a diminished understanding of their own, as well as of other people’s emotions, indicating a lowered perceived emotional intelligence [24].

These findings are of considerable interest in the context of this study, as the capacity to project oneself into someone else’s perspective is inherent in interpretation of sarcasm. Central to sarcastic remarks is the discrepancy between the sentence’s literal meaning and the meaning the speaker actually intends to convey [25]. Imagine that Tom asks Mary how the dinner with her mother went, and Mary replies “*We had cod*. *It was absolutely delicious*”. If it is obvious to both Tom and Mary that Mary hates fish, Tom will notice the discrepancy between the statement and such background, contextual information, and will likely interpret Mary’s answer as sarcastic. By contrast, if Tom does not have access to the relevant background knowledge, he will probably miss Mary’s sarcastic intent. A substantial body of research demonstrates that the ability of adults to infer the addressee’s interpretation of ironic statements is flawed by egocentric biases [26,27,28]. In these studies, participants are provided with privileged contextual information (e.g. Mary's dislike of fish) and have to gauge whether the target utterance will be perceived as sarcastic or not by an addressee who is lacking this contextual knowledge (e.g. who was not privy to Mary’s culinary preferences). Results indicate that participants make more judgmental errors in perspective-inconsistent trials, that is, when their own information state is different from the utterance’s addressee. In other words, they judge the utterance to be sarcastic or not from their own egocentric perspective, and this in spite of the incompatibility of their interpretation with the addressee's perspective. Participants are also slower in perspective-inconsistent than in perspective-consistent trials, which is expected if they adopt the addressee's point of view by initially anchoring on their own perspective then progressively adjusting to accommodate the point of view of the other [26,29,30].

The main hypothesis tested in the present study is that sleep deprivation should impair perspective-taking during sarcastic interpretation. In other words, sleep-deprived participants should be slower and less accurate than sleep-rested participants in detecting sarcasm, especially in perspective-inconsistent situations. Envisaging other people's minds has an obvious executive component [31,32,33]. Working memory and cognitive flexibility are necessary to hold multiple perspectives in mind, and inhibitory control is required to suppress irrelevant ones [34,35]. Inhibitory control is especially important in the perspective-inconsistent conditions of our Sarcasm Detection Task. Executive functions are heavily (although not exclusively) subtended by the functional integrity of the prefrontal cortex (for a review see [36]), a region attuned to acute sleep loss [37,38,39]. The current literature on the potential effects of sleep deprivation on executive function yielded mixed results, which are open to various interpretations (for a review see [4]). According to the “state instability” theory [40], the decline in performance outcomes after sleep deprivation is not necessarily due to deficits in executive functions. Instead, it may be partly explained by impairment in attention. Attention being a key factor in performance in many other cognitive processes, sleep loss would alter global performance with secondary or little effect on executive functions *per se* [41]. Inconsistencies in the literature studying executive functioning in sleep deprived conditions might reflect the use of tasks that did not clearly distinguish between executive and non-executive components, in particular attention-related task components [42,43]. To partially address this issue, participants in the present study were additionally administered vigilance (psychomotor vigilance) and inhibition (Stroop) tasks, allowing to assess the impact of these two functions on sarcasm detection in a sleep deprivation condition.

If sleep deprivation affects negatively the perspective-taking process supporting sarcasm comprehension, as our main hypothesis suggests, sleep-deprived subjects could rely more heavily on other types of cues. The aforementioned studies on perspective integration in sarcasm processing based their methodology on either written [27,28,29] or monotonously uttered target sentences [26]. Other studies investigating the role of intonation concluded that complementary to contextual information, prosodic cues can help disambiguating the speaker’s intent [44,45]. An arising question is therefore whether sleep-deprived subjects would rely as much as sleep-rested ones on this additional source of information or whether its processing results in an increased cognitive load.

## Materials and Method

### Participants

**Fig 1:**
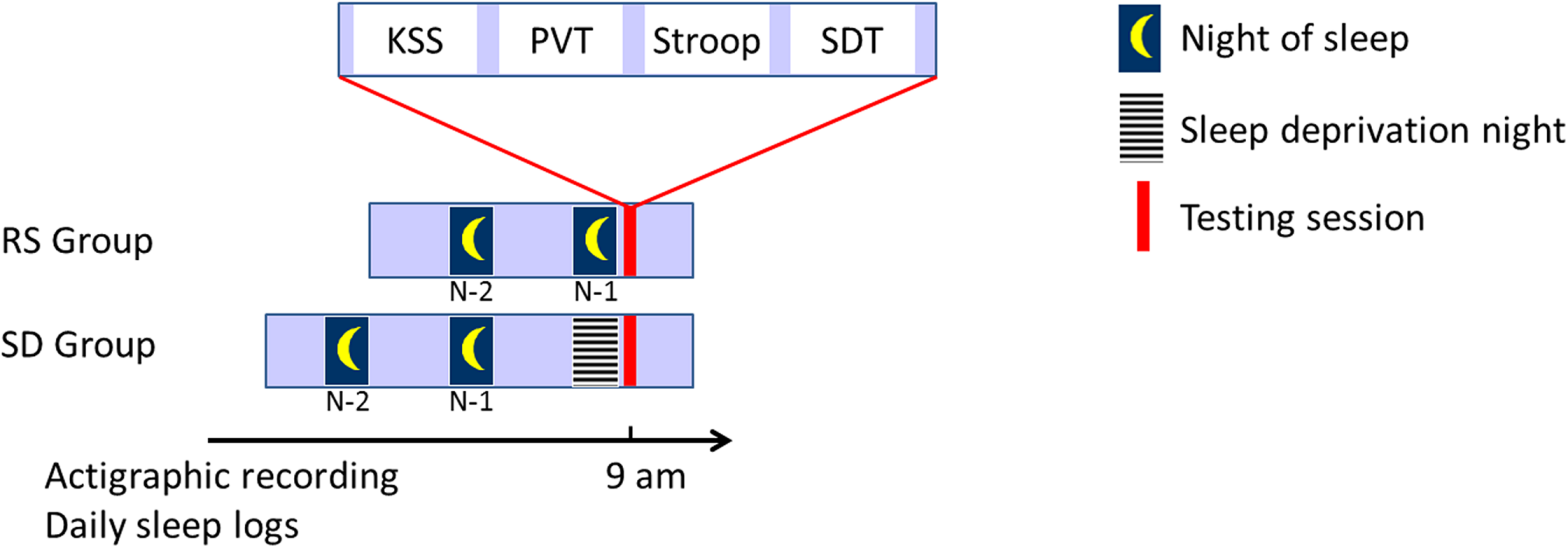
Experimental design. Regular Sleep group (RS), Sleep Deprived group (SD), Karolinska Sleepiness Scale (KSS), Psychomotor Vigilance Task (PVT), Stroop Task (Stroop), Sarcasm Detection Task (SDT), the night (N-1) and two nights (N-2) preceding the experimental session (RS: task administration; SD: sleep deprivation night and task administration).

The present study was part of a larger project investigating the effects of sleep deprivation on memory consolidation and resistance against interference. Thirty healthy participants gave written informed consent to take part in this study, which received approval from the ethics committee of the Université libre de Bruxelles, Belgium. The regular sleep group (RS) consisted of 13 women and 2 men (mean age and standard deviation [std], 22.4 ± 2.67), and the sleep deprivation group (SD) consisted of 11 women and 4 men (mean age and std, 21.13 ± 1.64). Participants met the following criteria: native French speakers, non- or moderate smokers (< 10 cigarettes per day), no sleep disorders (Pittsburgh Sleep Quality Index total score ≤ 5; [46]), no mood disorders (Beck’s Depression scale < 7; [47]), and intermediate or neutral chronotype (Horne and Ostberg’s Morningness-Eveningness Questionnaire [48]: range 37–69). Participants were asked to maintain regular sleep patterns during the two days before the experimental session, and to refrain from drinking alcohol or stimulant drinks (e.g. caffeine, energizers…) before the testing session and during the sleep deprivation period. The regularity of sleep habits was monitored using daily sleep logs for the two nights preceding the experimental session (i.e. 2 nights preceding tasks administration in the RS Group, 2 nights before the sleep deprivation night and subsequent tasks administration in the SD group; see Fig 1). Actigraphic recordings (wGT3X-BT, ActiGraph, Pensacola, Florida, USA) during the same periods were used to control the accuracy of self-reported sleep logs. Participants received monetary compensation upon the completion of the study.

### Materials

#### Vigilance and sleepiness measures

Vigilance and sleepiness were assessed using the 10-minute Psychomotor Vigilance Task (PVT; [49]) and the Karolinska Sleepiness Scale (KSS; [50]), respectively. In the PVT, participants were instructed to press a key as fast as possible whenever a millisecond countdown appeared in the middle of the computer screen. Stimuli were randomly presented with an inter-stimuli interval ranging from 2 to 10 seconds. The KSS is a self-report 9 points Likert scale, with 1 as the anchor for “not sleepy at all” and 9 for “very sleepy/nearly asleep”.

#### Personality Questionnaires

As explained above, a crucial feature of our study is that participants are required to adopt another person’s perspective to assess sincere or sarcastic statements. To ensure that potential differences between RS and SD groups in the Sarcasm Detection Task are not due to personality traits rather than to the transient factor “sleep deprivation”, empathy and perspective taking abilities were controlled using two personality questionnaires. The Interpersonal Reactivity Index (IRI; [51]) is a self-report questionnaire tapping four different aspects of this multidimensional concept: perspective taking, fantasy, empathic concern, and personal distress. Participants marked each of these aspects on a five points Likert scale ranging from “does not describe me well” to “describes me very well”. The participants’ Empathy Quotient was also measured using the Baron-Cohen & Wheelwright’s [52] eponymous questionnaire. The Empathy Quotient is a self-reported questionnaire using a 4-point scale ranging from “agree strongly” to “disagree strongly” on 60 statements, designed to assess subject’s cognitive and affective perspective taking.

#### Stroop task

To measure cognitive inhibition, a bimodal Stroop task was used (adapted from [53]). In this task, participants had to decide if the color word heard in the headphones (“red”, “yellow”, “blue” or “green” in French) was identical (i.e. a correct trial) or not (i.e. an incorrect trial) to the color of the ink of a written word displayed on the screen (i.e. a classical Stroop stimulus). Participants had to press, as fast as possible, “M” on the keyboard with the right index if it was a correct trial and “Q” with the left index if it was an incorrect one. As in a classic Stroop task, congruent, neutral and incongruent Stroop conditions were used (for a review see [54,55]). The congruent condition consisted of a color word inked in its own color (e.g. color word RED displayed in red), the incongruent condition consisted of a color word inked in any of the four colors, other than the one to which it referred (e.g. color word RED inked in green) and the neutral condition consisted of a neutral word (French words matched to color words for the number of letters, syllables and frequency of use; CHEF [chief], COURT [short], LIEUX [locations], DOUX [sweet]) inked in any one of the four colors (e.g. word CHEF displayed in red). Words were displayed at the center of the screen in Time New Roman, Bold, size 72 font. Participants completed three blocks of randomly ordered 123 trials, including 3 warm-up trials, 40 congruent, neutral and incongruent trials. Half of the trials were correct trials and half of them followed a correct one. Stimuli were displayed until response. The inter-stimuli interval was 500 ms. The target color was never the target or the distractor of the following trial to avoid proactive interference effects.

#### Sarcasm task

**Fig 2:**
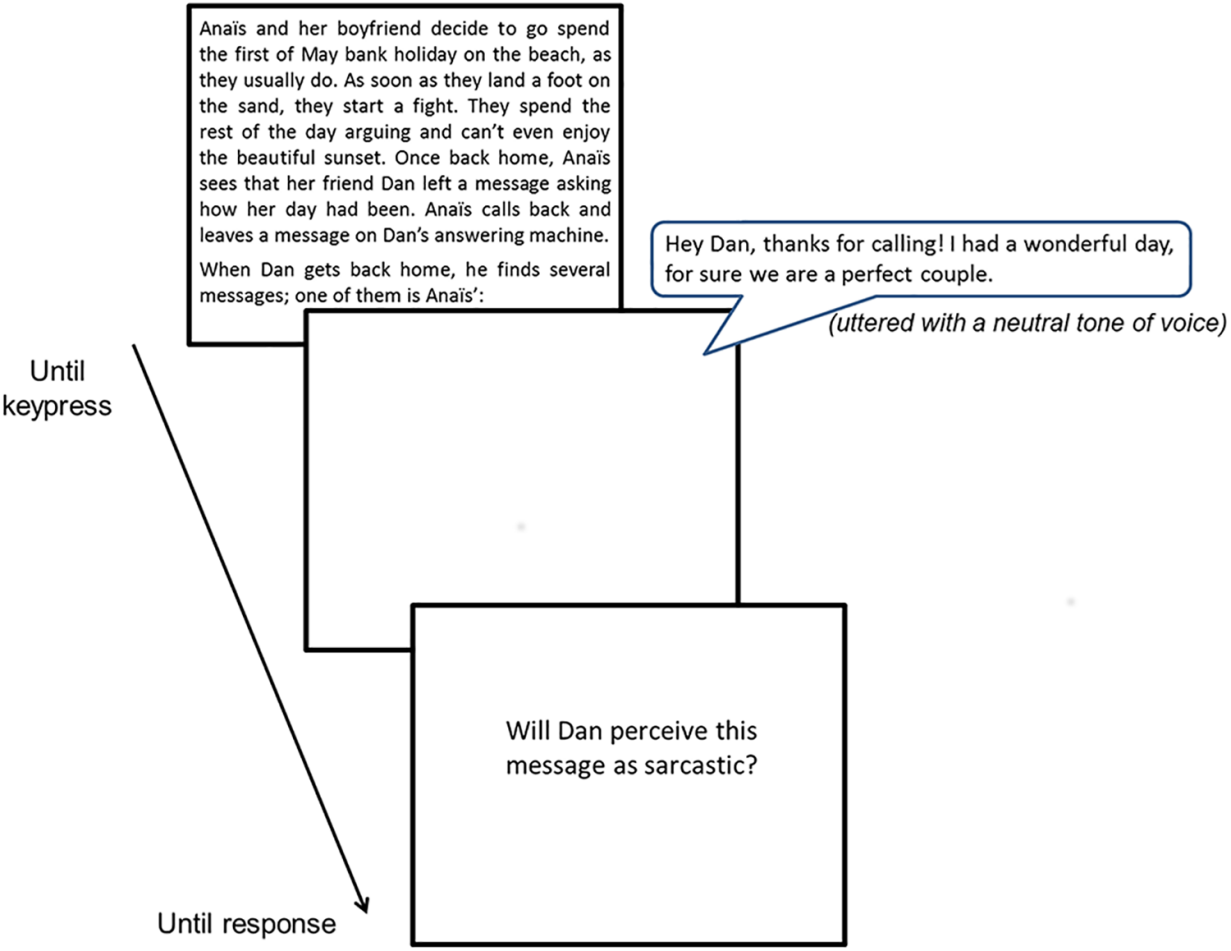
Sarcasm Detection Task: the course of a trial. All trials start with the written presentation, on the computer screen, of an event in the character *Anaïs Reton*’s life; the text remains on screen until the participant presses a key signalling that he finished reading the story. Voicemail messages are then auditorily delivered and directly followed by the question “Will he (or she) perceive this message as sarcastic?”. (Correct answer for this example is *No*). The next trial began once the participant had answered the question by pressing the corresponding key.

The task was translated in French and adapted from Epley et al. [26] with some scenarios inspired by Kreuz, Kassler, Coppenrath, & Allen [56] and Pexman & Zvaigzne [57]. First, participants had to read the description of an event in the life of the character *Anaïs Reton* on a computer screen (= the scenario). To avoid potential biases due to differences in reading speed between the RS and SD groups, the text remained on screen until the participant pressed a key signalling that he finished reading the story. In a second step, intentionally ambiguous voicemail messages left by one of the friends on the other's phone were delivered auditorily (i.e. the target sentence). Oral messages were directly followed by the written question “Will he (or she) perceive this message as sarcastic?” displayed on the computer screen (see Fig 2). Participants had to decide, as fast as possible, whether the message would be interpreted by the addressee as sincere or as sarcastic, by pressing the corresponding key (Yes [key "K"] / No [key "L"]). Eighteen scenarios and their associated target sentences were presented to the participants. Prior to the test phase, subjects were given a definition and an example of sarcasm, and were trained on a first scenario.

Twelve scenarios were designed, each with two versions. In the first version, the event turns out positive, and the correct interpretation of target utterance is a literal one (Literal scenarios: L). Moreover, in such literal scenarios, there is no discrepancy between the participant’s and the addressee’s perspective. For example:

Anaïs wants to go to the movies with friends. She hesitates between two films, one that has got good reviews and one that is coming out that night. Her friend Joan has a look at the options and says: “Is Leonardo Di Caprio in the one that is coming out tonight? Let’s go for that one. His movies are always great”. At the last minute Joan remembers that she has an essay due on the next day and she cancels the appointment. Anaïs and the others go to the movie theatre anyway. The movie is very good, one of the best they have ever watched. Later that night, Joan comes back from the library and sees she has received a message from Anaïs: **“Joan, I’ll keep following your advice on movies, the last Di Caprio was actually great!”**

The second version of the scenario had a bad ending. From the participant’s point of view this scenario was compatible with a sarcastic interpretation of the target utterance. However, the scenario was designed in such a way that the addressee of the voice message was not in possession of sufficient contextual information to interpret the target as sarcastic. In other words, the message can be sarcastic from the participant’s but not from the addressee’s perspective (Sarcastic egocentric: SE). For example:

Anaïs wants to go to the movies with friends. She hesitates between two films, one that has got good reviews and one that is coming that night. Her friend Joan has a look at the options and says: “Is Leonardo Di Caprio in the one that is coming out tonight? Let’s go for that one. His movies are always great”. At the last minute Joan remembers that she has an essay due on the next day and she cancels the appointment. Anaïs and the others go to the movie theatre anyway. The movie is very bad, one of the worst they have ever watched. Later that night, Joan comes back from the library and sees she has received a message from Anaïs: **“Joan, I’ll keep following your advice on movies, the last Di Caprio was actually great!”**

Since the question the participants have to answer concerns the addressee’s interpretation of the target utterance, providing the correct answer in the SE condition requires to inhibit their own knowledge of the context.

A third category comprises scenarios with a bad ending but in which the addressee of the voice message is in possession of enough contextual information to interpret the target utterance as sarcastic. In this case, the message is sarcastic from both the participant’s and the addressee’s perspectives (Sarcastic allocentric: SA). For example:

Anaïs is going on vacation to Barcelona. Her friend Clemence would also like to visit the city soon and asks Anaïs to tell her what she thinks of her hotel. Therefore Anaïs, once there, sends Clemence a post card saying: “Dear Clemence, you will love Barcelona at the one condition that you do not stay at the hotel we are in: it is dodgy, ugly and dirty! That excluded, it is all sun and party!” A few days later, Anaïs is back from her holidays and wants to call her friend to tell her about her trip. As she reaches the voice mail, she leaves a message: **“Hi Clemence, I’m back! I need to tell you about our hotel: a small and charming institution, and impeccably clean.”**

These 3 categories of scenarios are associated with a target sentence uttered with a neutral tone of voice. A fourth category comprises sarcastic messages similar to the SA category but uttered with a sarcastic intonation (SAI).

To ensure that the target sentences were recorded with the right tone of voice across conditions, fifteen volunteers, who did not take part to the experiment, were asked whether the target sentences, delivered without their surrounding context, were uttered with a sarcastic intonation or not. All the target utterances used in the L, SE and SA scenarios were scored as “sincere statement” by more than 85% of the participants; the 3 target utterances used for the SAI scenario were scored as sarcastic by 100% of the participants.

Two lists of scenarios were created so that each participant only encountered six L scenarios and six SE scenarios once in one of its versions. Three SA scenarios and three SAI scenarios were added to the two lists. Both lists had therefore those last six in common. The fifteen L, SE and SA items appeared first, in a randomized order, with never more than four stories of the same valence in a row. To investigate the specific effect of sarcastic intonation, both lists ended by the three SAI scenarios. Since the addition of a prosodic cue may create an altering effect, which can spread to the following trials, these three scenarios were not randomized with scenarios associate with a target sentence delivered on a monotonous tone.

**Fig 3:**
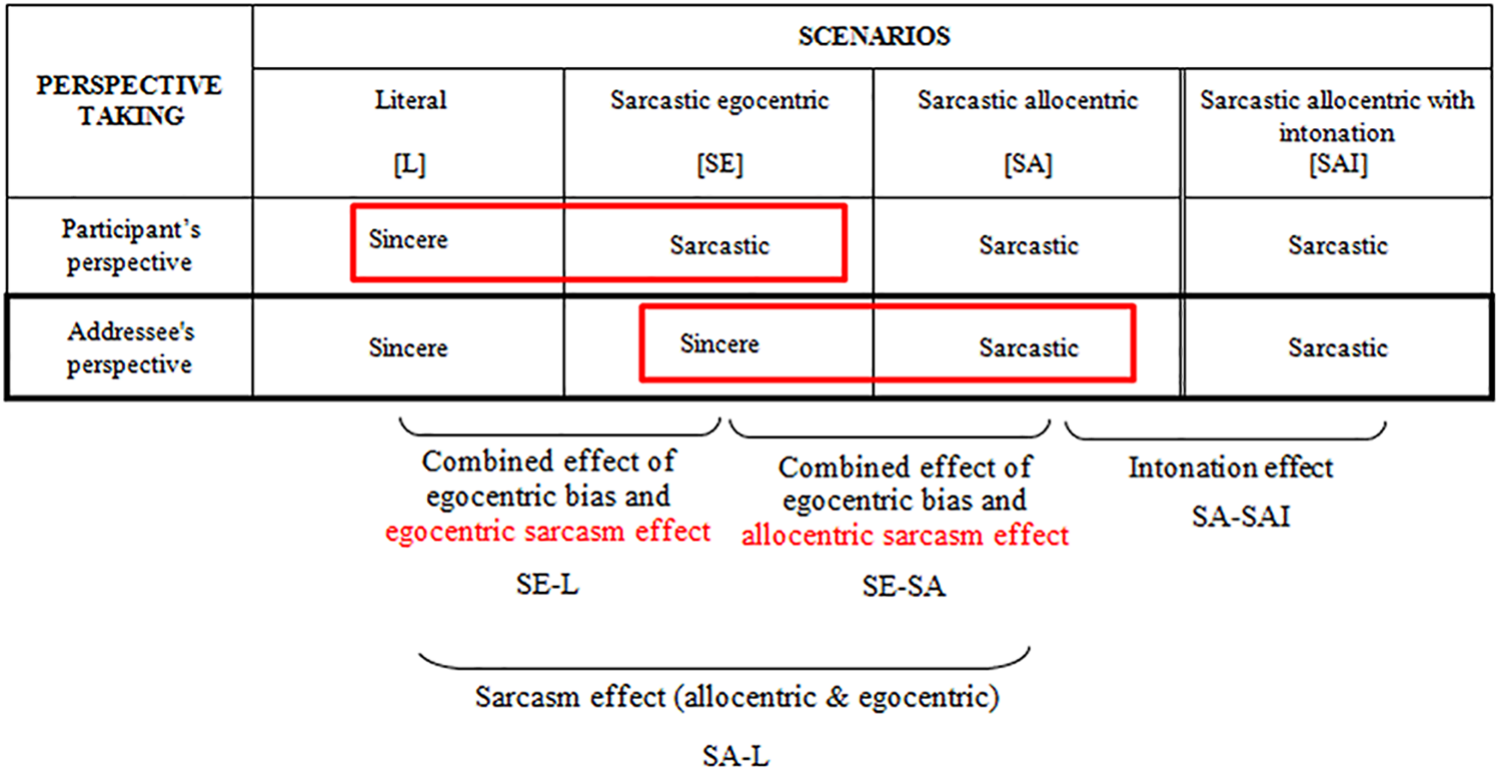
Sarcasm Detection Task.

To sum up, 18 messages (see S1 Text) were delivered on a monotonous tone and subdivided in three categories (see Fig 3): literal (L, n = 6) or sarcastic but the addressee is (SA, n = 3) or is not (SE, n = 6) in possession of enough contextual information to be able to interpret it as sarcastic. A fourth category (SAI, n = 3) comprised sarcastic messages similar to SA category but uttered with a clearly sarcastic tone. Comparisons between these four types of scenarios make it possible to isolate the *sarcasm* effect, the *intonation* effect, the combined effects of *egocentric bias and egocentric sarcasm*, and the combined effects of *egocentric bias and allocentric sarcasm* (Fig 3).

The *sarcasm* effect is obtained by subtracting performance in the literal from the sarcastic allocentric scenarios (SA-L), showing whether participants are better (errors rates or reaction times) at processing sarcastic vs. sincere statements. Since literal scenarios are sincere from both the participant’s and the addressee’s point of view and the sarcastic scenarios are sarcastic from both points of view, their comparison should reveal an allo- and egocentric sarcasm effect.

The difference between sarcastic egocentric and literal scenarios (SE-L) evidences a combined effect of egocentric bias and egocentric sarcasm. The egocentric sarcasm effect is defined as participants' failure to completely ignore their own privileged knowledge and to adopt the message addressee’s perspective. Recall that while in both scenarios the target utterance is a sincere statement from the addressee's point of view, in the SE scenarios participants must inhibit their own contextual knowledge to adequately infer the addressee's interpretation. This egocentric bias is combined here with an egocentric sarcasm effect since, from the participant’s point of view, literal scenarios are sincere whereas sarcastic egocentric scenarios are sarcastic.

The combined effect of egocentric bias and the allocentric sarcasm effect allows a comparison between sarcastic allocentric and sarcastic egocentric items. Both scenarios are sarcastic from the participant’s point of view. However, the later, but not the former, are literal statements from the addressee's point of view. This combined effect of egocentric bias and the allocentric sarcasm effect is computed as the difference between sarcastic egocentric and sarcastic allocentric scenarios (SE-SA).

Finally, to investigate whether adding a prosodic cue to sarcastic statements leads to better performances—namely the presence of an ‘intonation effect’—the difference between performances in the sarcastic allocentric scenarios and sarcastic allocentric scenarios with sarcastic intonation (SA-SAI) was computed.

### Procedure

Participants assigned to the SD group were kept in the laboratory from 18:00 to 10:00 next day under the constant supervision of three experimenters. During the sleep deprivation period, SD participants were asked to remain seated most of the time and to engage in quiet activities (e.g. reading or watching movies). Free water and regularly offered isocaloric meals were available. The 10-minute Psychomotor Vigilance Task (PVT; [49]) and the Karolinska Sleepiness Scale (KSS; [50]) were administered hourly to estimate the evolution of objective and subjective vigilance levels, respectively, through the SD night. Participants assigned to the RS group came to the laboratory in the morning for the testing session after a normal night of sleep at home. At 9:00, all participants filled in the KSS and performed the PVT followed by the Stroop task and the Sarcasm Detection Task. The regularity and quality of sleep at home was controlled (questionnaires and actimetry) for the two nights preceding the experimental manipulation. An overview of the experimental design is illustrated Fig 1.

### Statistical analyses

Statistical analyses were performed using Statistica 7.0 (Statsoft Inc., Tulsa, OK). Data are expressed as mean ± SD. Significance level was set at p < 0.05 (two-tailed). Post-hoc tests in ANOVAs were performed using Tukey’s correction for multiple comparisons.

In the PVT task, tonic alertness was computed using the Reciprocal Response Time (RRT = mean 1/RTs) and the variability of valid RTs (RTs ≥ 100 ms). RRT was shown the most sensitive PVT outcome metric to highlight the impact of sleep loss on alertness [58]. To calculate the RRT, each RT (ms) was converted in seconds and then reciprocally transformed. The average of all transformed values was then computed [59]. RT variability was computed as the difference between the 10% slowest and the 10% fastest RTs [40]. A lower Reciprocal Response Time and a higher RT variability indicate poorer performance.

The impact of sleep deprivation in the Sarcasm Detection Task was computed on the percentage of judgmental errors and on RTs. To facilitate comparisons with prior studies [26], we first computed the global impact of sleep deprivation on stimuli uttered with neutral intonation (L, SE, SA). This analysis aims at determining whether sleep-deprived participants are slower and less accurate than sleep-rested participants in detecting sarcasm, especially in perspective-inconsistent situations (i.e. SE). Secondly, to investigate whether sleep-deprived subjects rely as much as sleep-rested ones on sarcastic intonation or whether its processing results in an increased cognitive load, a separate analysis was carried out on Sarcastic Allocentric stimuli uttered with a neutral vs. a sarcastic intonation (SA vs. SAI).

## Results

### Descriptive statistics

Participants in the RS and SD groups did not differ according to age (t(28) = -1.57, p = 0.13), verbal intelligence (Mill Hill Vocabulary Scale; [60]) (t(28) = -0.16; p = 0.87), empathy quotient (t(28) = -0.52; p = 0.61) or the four subscales of the Interpersonal Reactivity Index (all ps > 0.26, see Table 1).

**Table 1:** Means, standard deviations and group comparison.

Measures	Group					Student’s t- test
	SD		RS			
	N = 15		N = 15			
	(4 males)		(2 males)			
	Mean	SD	Mean	SD	*T*	p-value
Age	21.13	± 1.64	22.40	± 2.67	-1.57	0.13
Mill Hill score	33.27	± 2.74	33.53	± 5.82	-0.16	0.87
Empathy quotient	41.87	± 9.98	43.47	± 6.58	-0.52	0.61
IRI						
Perspective taking	17.67	± 4.70	17.33	± 4.32	0.20	1^a^
Fantasy	20.33	± 4.86	21.33	± 2.89	-0.68	0.26^a^
Empathic concern	20.60	± 3.56	20.13	± 4.44	0.32	1^a^
Personal distress	15.67	± 4.34	16.27	± 4.74	-0.36	1^a^

Notes: IRI = Interpersonal Reactivity Scale, ^a^ corrected for multiple comparisons.

### Sleep and vigilance variables

Concordance between actimetric recordings and self-reported sleep logs was controlled by visual inspection. Due to a technical failure with one actimeter, concordance could not be verified for one participant. A mixed measures ANOVA conducted on mean sleep duration with within subject factor Night (N-2, N-1 before the experimental session) and between subject factor Condition (RS vs. SD) failed to reveal a main effect of Condition (F1, 28 = 0.14; p = 0.71) or Night of sleep (F1, 28 = 0.68; p = 0.42). A marginally significant interaction between Night and Condition (F1, 28 = 3.7; p = 0.06) was not supported by Tukey’s post-hoc comparisons (RS group: N-2 = 8h15min ± 1h05min, N-1 = 7h38min ± 1h18min, SD group: N-2 = 7h58 ± 59min, N-1 = 8h11min ± 1h13min, all ps > 0.23).

During the SD night, self-reported sleepiness (KSS) increased over successive hours (from well awake [2.60] to very tired [6.67]; F15, 210 = 24.44; p < 0.001). A similar analysis conducted on PVT data (RRT and RT variability) disclosed smaller RRT (from 3.05 ± 0.26 at 18:00 to 2.50 ± 0.24 at 9:00, F15, 210 = 20.33, p < 0.001) and higher RT variability (from 121.05 ± 41.62 at 18:00 to 314.30 ± 184.23 at 9:00, F15, 210 = 2.67, p < 0.001) across the SD night.

Before the testing session (9:00), participants in the SD group reported more sleepiness (KSS = 6.67 ± 1.59; t(28) = 4.71; p < 0.001) and presented lower vigilance level and higher RT variability (RRT = 2.50 ± 0.24, t(28) = -5.21, p < 0.001, RT variability = 314.30 ± 184.23, t(28) = 3.87; p = 0.001) than the RS group (KSS = 4.00 ± 1.51, RRT = 3.06 ± 0.34, RT variability = 125.04 ± 44.96).

### Stroop Task

Two participants in the SD group presented outlier results at the Stroop task (more than 3 standard deviations from participants’ mean on RTs or on errors) and were excluded from the Stroop analyses. A mixed measured ANOVA on Reaction Times (RTs) with within factor Congruency (Congruent, Incongruent, Neutral trials) and between factor Group (RS vs. SD) disclosed a main Congruency effect (F2, 52 = 21.35; p < 0.001). Tukey post-hoc analysis showed that participants responded faster in congruent (716 ± 257 ms) than in neutral (817 ± 257 ms, p = 0.003) and incongruent (909 ± 325, p < 0.001) trials, and were slower in incongruent than in neutral trials (p = 0.009). We also observed a trend for slower responses in the SD than in the RS condition (899 ± 362 ms vs 728 ± 152 ms; F1, 26 = 3.08; p = 0.091). The interaction between Congruency and Group was non significant (F2, 52 < 0.001; p = .99).

A mixed model ANOVA was computed on the percentage of errors with within factor Congruency (Congruent, Incongruent, Neutral trials) and between factor Group (RS vs. SD). This analysis yielded a significant Congruency effect (F2, 52 = 56.79; p < 0.001). Tukey post-hoc analysis showed that participants made more errors in incongruent (18.80 ± 11.07) than in neutral (5.60 ± 6.02) or congruent (3.57 ± 3.82, all ps < 0.001) trials, neutral and congruent trials did not differing (p = 0.4) from each other. Main effect of Group and the interaction Group x Congruency did not reach significance (all Fs < 1).

### Sarcasm Detection Task

#### Judgmental errors on messages with neutral intonation

**Fig 4:**
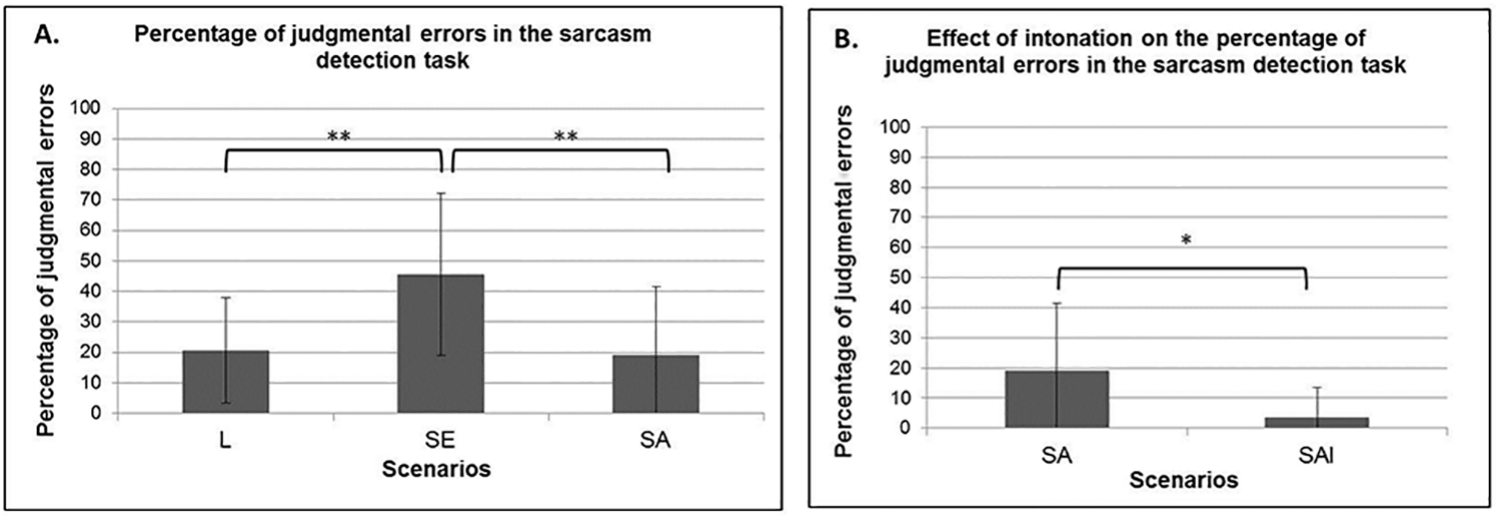
A. Percentage of judgmental errors for literal (L), sarcastic egocentric (SE), sarcastic allocentric (SA) scenarios. B. Percentage of judgmental errors for sarcastic allocentric (SA) and sarcastic allocentric with intonation (SAI) scenarios. * = p ≤ .01, ** = p ≤ 0.005; *** = p < .001.

A mixed model ANOVA on the percentage of judgmental errors with within factor Scenario (L, SE, SA) and between factor Group (SD vs. RS) disclosed a significant main effect of Scenario (F2, 56 = 14.93; p < 0.001). Tukey post-hoc analyses revealed that participants made more judgmental errors in sarcastic egocentric (45.56% ± 26.60%) than in literal (20.56% ± 17.33%) and sarcastic allocentric (18.89% ± 22.63%, all ps < 0.001, Fig 4A) scenarios. Neither the main effect of Group nor the interaction Group x Scenario were significant (all Fs < 1).

#### Intonation effect on judgmental errors

To investigate the specific effect of sarcastic intonation, a separate analysis was carried out on the percentage of judgmental errors, with within factor Scenario (SA and SAI) and between factor Group (SD vs. RS). The main effect of Scenario was significant with fewer judgmental errors in sarcastic allocentric scenarios uttered with a sarcastic tone (3.33% ± 10.17%) than in sarcastic allocentric scenario uttered with a monotonous tone [18.89% ± 22.63%, F1, 28 = 11.93; p = 0.002, Fig 4B]. Neither the main effect of Group nor the interaction Group x Scenario reached significance (all Fs < 1).

#### Reaction times on messages with neutral intonation

**Fig 5:**
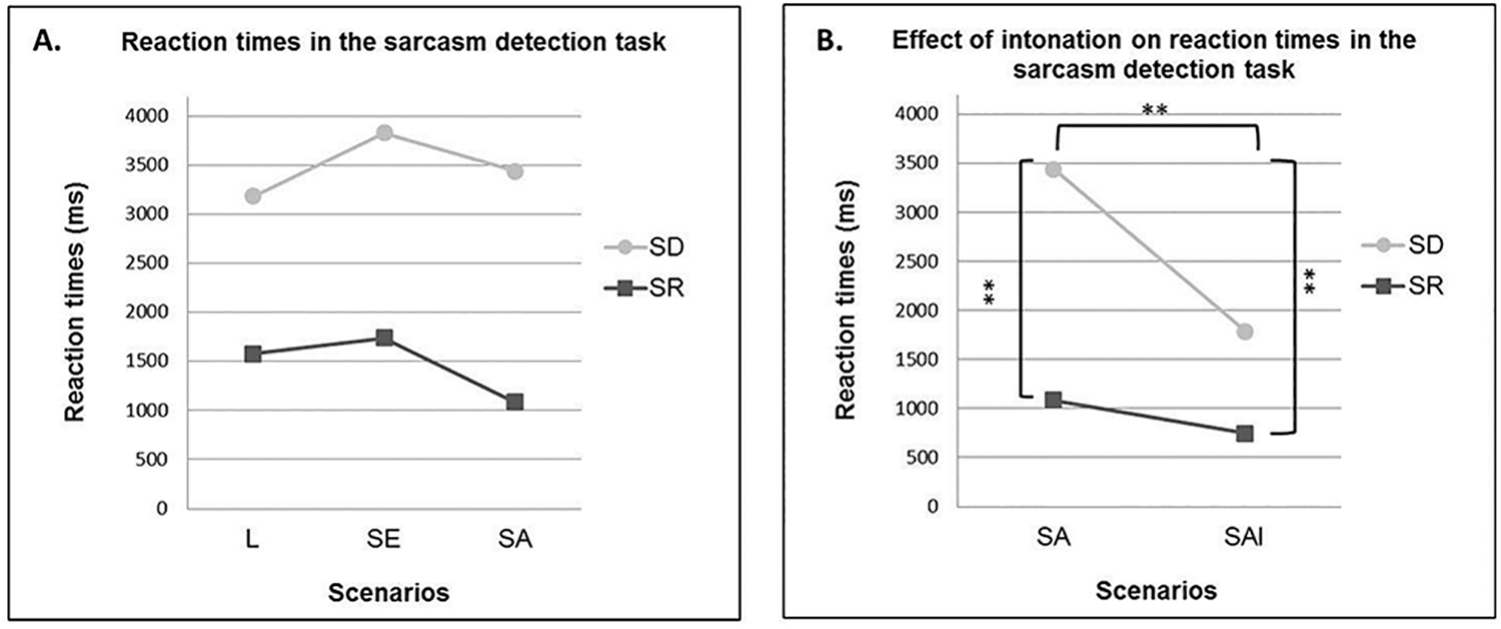
A. Reaction times (ms) in the Sarcasm Detection Task. Literal (L), Sarcastic Egocentric (SE), Sarcastic Allocentric (SA) scenarios. B. Reaction times (ms) in the Sarcasm Detection Task. Sarcastic Allocentric (SA) and Sarcastic Allocentric with Intonation (SAI) scenarios. The right bracket connects the SD group in the SA condition to the SR group in the SAI condition. The SD vs. SR groups in the SAI condition are connected by the left bracket. **p < 0.005

A mixed model ANOVA on RTs with within factor Scenario (L, SE, SA) and between factor Group (SD vs. RS) yielded a significant main effect of Group, indicating a global slowdown after SD (3484 ± 1839 ms) as compared to RS (1466 ± 1037 ms, F1, 28 = 18.60; p < 0.001). There was also a marginal trend for a main effect of Scenario (F2, 56 = 2.49; p = 0.09). Since the F-value was > 1 despite a p > .05 [61], we computed post-hoc comparisons for informational purpose. Tukey post-hoc analyses indicated a trend for slightly faster RTs in sarcastic allocentric (2260 ± 1926 ms) than in sarcastic egocentric scenarios (2785 ± 1954 ms; p = 0.09), suggesting a possible combined effect of egocentric bias and allocentric sarcasm effects (Fig 5A). No main Sarcasm effect was evidenced, as RTs were similar for positive and sarcastic scenarios (p = 0.88) and there was no combined effect of egocentric bias and egocentric sarcasm (p = 0.24). Finally, the interaction Group x Scenario was non significant (F2, 56 = 1.17; p = 0.32).

#### Intonation effect on reaction times

A similar analysis on RTs with within factor Scenario (SA vs SAI) and between factor Group (SD vs. RS) disclosed a main effect of Group (F1, 28 = 19.99; p < 0.001), a main effect of Scenario (F1, 28 = 15.45; p < 0.001) and a Group x Scenario interaction (F1, 28 = 6.79; p = 0.01). Tukey post-hoc analyses showed slower RTs for sarcastic allocentric scenarios in the SD group (3831 ± 1961 ms) in comparison to RS group (1082 ± 931 ms). RTs for sarcastic allocentric scenarios in the SD were also slower than sarcastic allocentric scenarios uttered with intonation in both the SD (1784 ± 1145 ms) and RS groups (747 ± 458 ms; all ps < 0.001, Fig 5B). All other comparisons were non significant (all ps > 0.24).

#### Attentional modulations of the effect of SD on reaction times

To investigate whether the effect of SD on RTs was better explained by attentional modulations, correlations between mean RTs in the Sarcasm Detection Task for all categories of stimuli (L, SE, SA, SAI) and attentional variables affected by sleep deprivation (PVT RRT and RT variability) were computed. After correction for multiple comparisons, RTs in the Sarcasm Detection Task were found negatively correlated with RRT (r = -0.52, p = 0.006) but not with PVT variability (r = 0.364, p = .10). To explore further this association, we conducted a mediation analysis [62, 63] aimed at controlling whether RTs differences between RS and SD groups in the Sarcasm Detection Task are mediated by these attentional components. A simple mediation model was computed with Group (RS vs. SD) as the independent variable, RTs in the Sarcasm Detection Task as the dependent variable, and the RRT in the PVT as the mediator variable. The significance of this indirect effect was tested using a bootstrapping procedure. Unstandardized indirect effects were computed for each of the 10,000 bootstrapped samples, and the 95% confidence interval was computed by determining the indirect effects at the 2.5th and 97.5th percentiles. The bootstrapped unstandardized indirect effect was -236.75, and the 95% confidence interval ranged from – 932.94 to 821.48. The indirect effect was thus not statistically significant (Sobel test’s p = 0.549). Therefore, this mediation analysis indicates that differences in RTs between RS and SD groups in the Sarcasm Detection Task are not completely mediated by a modulation of vigilance.

#### Impact of sleep deprivation on specific effects and biases

The preceding analyses evidence an impact of sleep deprivation on each type of scenarios separately. To investigate the impact of sleep deprivation on the sarcasm effect and on the two combined effects (the combined effect of egocentric bias and egocentric sarcasm, the combined effect of egocentric bias and allocentric sarcasm), Student’s t-tests (corrected for multiple comparisons) were computed on RTs. The sarcasm effect (SA-L) did not differ between the SD and RS groups (t(28) = 1.49; p = 0.45). Neither the combined effect of egocentric bias and egocentric sarcasm (SE-L) nor the combined effect of egocentric bias and allocentric sarcasm (SE-SA) were significantly different between groups (t(28) = 0.95; p = 1 and t(28) = -0.55; p = 1, respectively).

A separate analysis was computed on the intonation effect. Results indicate that the beneficial effect of intonation on RTs is greater in the SD (1653 ± 1699 ms) than in the RS group (335 ± 975 ms, t(28) = 2.61; p = 0.015).

Finally, correlations between the intonation effect on RTs and variables affected by sleep deprivation were computed. After correction for multiple comparisons, the intonation effect was negatively correlated with PVT RRT (r = -0.408; p = 0.05) but not with PVT variability or KSS (all ps > .15). Again, a mediation analysis [62] was conducted to test how differences between RS and SD groups in the intonation effect were mediated by this attentional component. A simple mediation model was computed with Group (RS vs. SD) as the independent variable, the intonation effect on RTs as the dependent variable, and the RRT in the PVT as the mediator variable. The bootstrapped unstandardized indirect effect was -402.56, and the 95% confidence interval ranged from -1710.21 to 678.02. The indirect effect was thus not statistically significant (Sobel’s test p = 0.436). It suggests that differences between RS and SD groups in the intonation effect are not mediated by a mere vigilance effect.

## Discussion

Detecting a speaker's sarcastic intention and adequately inferring whether his/her message will be perceived as sarcastic by the addressee is a complex process, which recruits intertwined cognitive functions such as inhibition, working memory, flexibility and attention. Given such a wide range of high-order processes, one may expect that sleep-deprived persons should fail in a Sarcasm Detection Task that involves perspective taking. Interestingly, our results show that sleep deprived (SD) participants end up being as accurate as participants having slept normally (RS), indicating that (fortunately) a night of sleep deprivation does not completely hinder one’s ability to interpret sarcasm. However, we also found that SD participants were significantly slower to adopt another person’s perspective. Such a result pattern, consisting in the absence of effect on accuracy but an effect on reaction time, has been found in a wide variety of tasks conducted after sleep deprivation. Indeed, one sleep deprivation night has been found to alter reaction times in a working memory task but not working memory scanning efficiency and resistance to proactive interference [43]. Similarly, despite slower RTs, visuospatial perception in a Judgment of line Orientation test [64] was intact as well as interference or facilitation to a classic [42] and emotional Stroop task [65]. In a study designed to investigate facial mimicry, a process thought to subtend the identification of others’ emotions, participants were asked to react to emotional pictures with facial muscles that were either congruent or incongruent to the valence of the stimulus. Interestingly, facial electromyographic data evidenced slower volitional facial reactions after sleep restriction in the absence of alteration in affective inhibitory control [66].

In our study, this overall slowdown in performance is in agreement with the “state instability” theory [41]. According to this theory, during sleep deprivation the interaction between the accumulating homeostatic pressure for sleep and the opposed drive to sustain alertness results in a fluctuation of sustained attention (i.e. longer RTs, increased RT variability, number of lapses and false starts). Nevertheless, mediation analyses show that blunted reaction times in the Sarcasm Detection Task cannot be fully explained by a mere attentional or motor slowdown as assessed by the Psychomotor Vigilance Task. The speed decrease observed in the Sarcasm Detection Task may thus reflect compensatory mechanisms supporting normative accuracy. In evolutionary terms, understanding, predicting and manipulating the behavior of others are all essential skills to ensure survival and reproduction [67] and are required in daily life to achieve successful social interactions. The importance of such abilities rooted in perspective-taking skills [68] could explain why sleep-deprived subjects tend to compute another person’s perspective accurately, even if it involves engaging in a time consuming process.

Egocentric biases were not more pronounced in the SD than in the RS groups, again suggesting that inhibition processes are relatively preserved after one night of sleep deprivation. Likewise, we did observe an overall increase in response time in the Stroop task in the SD group; however, the additional time needed in incongruent trials (i.e. when the color and the word did not match) compared to congruent trials did not change in comparisons with the RS group. The similar interference effect size observed in the Stroop task in the RS and SD groups gives some credence to the argument that extended wakefulness does not alter inhibition processes, in line with other studies [42,65,69].

Nevertheless, it would be interesting to disentangle the ability to inhibit one’s own point of view when asked to do so from the tendency to do it spontaneously. In our study, participants were explicitly asked to adopt the addressee’s perspective or, in other terms, to inhibit their own perspective. Whether sleep-deprived participants are more likely to spontaneously attribute their inner state of mind to others as compared to rested-participants also remains an open question that should be addressed in future studies. Further, our experimental design does not require switching from one perspective to another across trials, which is often the case in “real-life” situations. In a classic task-switching paradigm, where participants had to perform two different tasks in random succession, Couyoumdjian et al. [70] observed a deleterious effect of sleep deprivation on both accuracy and speed. Therefore, adding “Self perspective” trials to the current “Other’s perspective” trials could increase egocentric bias and potentially evidence flexibility impairment in sleep-deprived participants.

In daily life, we must adequately but also quickly infer others’ mental states. Interestingly, our results indicate that adding prosodic cues may compensate for the increased processing difficulties involved in sarcasm detection after sleep deprivation. The first explanation that comes to mind is that prosody has an alerting effect on participants as it creates contrasts [44]. The beneficial effects of prosody on reaction times might thus be more marked in the SD group simply because they are sleepier than the RS participants. Nevertheless, mediation analyses indicate that the difference between the RS and SD groups in the intonation effect is not fully mediated by alertness. Therefore, an alternative interpretation is that it merely results from the addition of supplementary cues allowing to disambiguate the speaker's communication intent, rather than from a specific effect of prosody. While sarcastic intent can be detected on the basis of intonation in the absence of contextual information [71,72] (except for dry sarcasm [73]), as well as through contextual information solely [26], people seem to process both in the presence of these two kinds of cues [45,71]. In our present study, the beneficial effects of tone of voice were even more pronounced in sleep-deprived participants indicating that they need more cues to counteract the overall slowdown in sarcasm detection. Further studies should investigate the respective effect of the addition of prosodic, body language and/or facial cues on processing time for sarcasms in RS and SD conditions.

An intriguing question that arises from this result is whether SD participants still process the contextual information when a prosodic cue is added. Although somewhat speculative, it is conceivable that sleep-deprived participants focused solely on prosodic cues to gauge the addressee’s perspective in order to avoid to retrieve contextual information from working memory, a highly time-consuming process after sleep deprivation [43]. Indeed, processing contextual cues is more complex than processing prosody. For instance, infants are able to understand sarcasm from prosodic cues at about 5 years of age, whereas their skills in interpreting sarcasm from contextual cues appears only later around 7 years old [74]. In our study, in order to respond correctly in the Sarcasm Detection Task, participants had to store in working memory what the agents of the scenario said and did. Thus, when the target sentence was delivered, participants had to compare the information from the target sentence with contextual information stored in working memory, in order to detect a discrepancy between the literal meaning of the sentence and the speaker’s intended meaning. When discrepancy is detected, participants still need to verify that the addressee possesses enough contextual knowledge to infer that the later will interpret it as sarcastic. This last step also requires retrieving information stored in working memory. Since working memory is dampened after acute sleep loss due to the overall slowdown [43], one could expect that when the target sentence is uttered with a sarcastic tone of voice, the sleep group would still integrate the linguistic (statement content), paralinguistic (e.g. vocal prosody) and contextual information before answering [45], whereas the sleep-deprived group would ground their decision on the prosodic cues only, a process less attuned to sleep loss. This compensation strategy would allow them to be as fast as the sleep group to infer the addressee’s perspective but can lead to misapprehension of some forms of sarcasm such as dry sarcasm [73].

Although the present data suggest that sleep deprivation might damage the flow of social interactions by slowing perspective-taking processes, our study also presents some limitations. First, the use of 24-hr acute sleep deprivation protocol as the only manipulation does not allow to evidence sleep dose-response effects on social cognition, an effect that should be investigated in further studies. In this respect, studying the impact of longer sleep deprivation or chronic sleep restriction on sarcasm detection may yield valuable inputs. That said, a 24-hr sleep deprivation protocol presents ecological value, as it is closer to real world situations, where one generally does not stay more than one night awake. Furthermore, chronic sleep restriction (4 h and 6h of sleep per night for 14 days) was shown to impair cognitive performance to a similar extent than one night of total sleep deprivation [75]. Hence, we could expect a similar slowdown in sarcasm detection after a moderate sleep restriction repeated over several days. Second, although some studies using a Sarcasm Detection Task include more than fifty participants [26,45], administrating sleep deprivation to such a large sample was not feasible in the present study. The absence of a sleep deprivation effect on judgment accuracy in the Sarcasm Detection Task and on RTs for the specific effects and biases (i.e. the sarcasm effect [SA-L], the combined effect of egocentric bias and egocentric sarcasm [SE-L] and the combined effect of egocentric bias and allocentric sarcasm [SE-SA]) might be due to the fact that the study was not powered to detect differences in a social cognition task. However, given the effect size that we obtained, we would have needed a sample size ranging from n = 178 to 25190 to detect significant effects, which is largely above the sample size encountered in classic sarcasm detection studies [26,45]. Beside limited sample size, another potential limitation is the lack of gender balance within our two experimental groups, mostly constituted of women. Several studies found an overall effect of gender on reaction time tasks, with men being faster than women at baseline, as well as a specific effect of sleep deprivation with an increased gender effect after sleep deprivation [76,77]. According to Blatter et al. [76], men and women tend to adopt different strategies to tackle sleep debt: men tend to be as fast as possible whereas women tend to avoid false starts by blunting their reaction times. In the present study we had a large majority of women participants in both groups, which may explain why our main results are observed on processing speed. More men were also present in the SD than in the RS group, but their restricted number as compared to women participants is unlikely to have tweaked the results in the between-group comparisons. Considering Blatter et al. [76] findings, future studies should investigate potentially different response patterns between men and women in sarcasm detection on larger, gender-balanced samples. It should be noted, however, that studies on a potential gender effect in the use of sarcasm have reported mixed results [25,78] whereas the effect of gender on sarcasm detection remains, to the best of our knowledge, unexplored.

## Conclusion

To sum up, our study shows that sleep deprivation does not completely hinder the ability to interpret sarcasm, since SD participants are as accurate as RS participants in inferring whether the addressee will interpret an utterance as sarcastic, but slow down the process of gauging another people’s perspective. Mediation analyses show that blunted decision times after sleep deprivation are not fully explained by a generalized SD-related cognitive slowing. Rather, it might reflect a compensatory mechanism supporting normative accuracy level in sarcasm understanding. Adding prosodic cues compensate for increased processing difficulties in sarcasm detection after sleep deprivation, possibly through a strategic reallocation of cognitive resources in favouring a decision process based on prosodic cues only.

## Supporting Information

S1 Text Sarcasm Detection Task: experimental material translated from French. (DOCX)
