# Supplementary material for: Impact of Acute Sleep Deprivation on Sarcasm Detection
Source: PLoS One. 2015 Nov 4;10(11):e0140527. doi: 10.1371/journal.pone.0140527 (PMC4633173; doi:10.1371/journal.pone.0140527)
Supplement: S1 Text — (DOCX) [file pone.0140527.s001.docx]

## S1. Sarcasm Detection Task: experimental material translated from French

## Literal and sarcastic egocentric (L and SE)

### The movies

(SE)

Anaïs wants to go to the movies with friends. She hesitates between two films, one that has got good reviews and one that is coming out tonight. Her friend Joan has a look at the options and says: Is Leonardo Di Caprio in the one that is coming out tonight? Let’s go for that one. His movies are always great ». At the last minute Joan remembers that she has an essay due on the next day and she cancels the appointment. Anaïs and the others go to the movie theatre anyway. The movie is very bad, one of the worst they have ever watched. Later that night, Joan comes back from the library and sees she has received a message from Anaïs:

(L)

Anaïs wants to go to the movies with friends. She hesitates between two films, one that has got good reviews and one that is coming out tonight. Her friend Joan has a look at the options and says: Is Leonardo Di Caprio in the one that is coming out tonight? Let’s go for that one. His movies are always great ». At the last minute Joan remembers that she has an essay due on the next day and she cancels the appointment. Anaïs and the others go to the movie theatre anyway. The movie is very good, one of the best they have ever watched. Later that night, Joan comes back from the library and sees she has received a message from Anaïs:

**« Joan, I’ll keep following your advice on movies, the latest Di Caprio movie is great! »**

### The lunch

(SE)

Anaïs has to meet her friend Mike for lunch. Just before Anaïs leaves, Roger, a good friend living abroad, calls her. Roger will be in town for just a few hours and would like to take that chance to go for lunch with Anaïs. However, knowing that she won’t be able to reach Mike, Anaïs is very sorry but has to decline the offer. When arriving at the office, Anaïs gets a message from Mike saying: « Anaïs, I’m sorry but my meeting is going to be longer than expected and I won’t make it to lunch. I hope it is not too much of an inconvenience ». Anaïs calls back and leaves a message on Mike’s answering machine. After his meeting, Mike gets back to his office. There, he finds a message from Anaïs, saying:

(L)

Anaïs has to meet her friend Mike for lunch. Just before Anaïs leaves, Roger, a good friend living abroad, calls her. Roger will be in town for just a few hours and would like to take that chance to go for lunch with Anaïs. Anaïs would have liked to meet with her old friend one-on-one, but knowing she won’t be able to reach Mike to cancel the meeting, she invites Roger to join them at the restaurant. When arriving to the office, Anaïs gets a message from Mike saying: « Anaïs, I’m sorry but my meeting is going to be longer than expected and I won’t make it to lunch. I hope it is not too much of an inconvenience ». Anaïs calls back and leaves a message on Mike’s answering machine. After his meeting, Mike gets back to his office. There, he finds a message from Anaïs, saying:

**« Hi Mike, Thanks for letting me know about this last minute change, I happen to have a friend visiting and he was hoping to spend a little bit of time with me during lunch break ».**

### The day at the beach

(SE)

Anaïs and her boyfriend decide to go spend the first of May bank holiday on the beach, as they usually do. As soon as they set foot on the sand, they start fighting. They spend the rest of the day arguing and can’t even enjoy the beautiful sunset. Once back home, Anaïs sees that her friend Dan left a message asking how her day had been. Anaïs calls back and leaves a message on Dan’s answering machine.

When Dan gets back home, he finds several messages; one of them is Anaïs’:

(L)

Anaïs and her boyfriend decide to go spend the first of May bank holiday on the beach, as they usually do. It is one of the nicest moments they have ever spent together. They spend the day bathing, relaxing and eating. They finally go back home after enjoying the beautiful sunset. Once back home, Anaïs sees that her friend Dan left a message asking how her day had been. Anaïs calls back and leaves a message on Dan’s answering machine.

When Dan gets back home, he finds several messages; one of them is Anaïs’:

**« Hey Dan, thanks for calling! I had a wonderful day, we’re certainly a perfect couple».**

### The experiment

(SE)

Anaïs gets back from her classes and finds a message on her answering machine saying: Anaïs, I am Joan, I am a Master’s student and I am running a study on how people react to psychological experiments. I have received your name in a list of students who participated in a study. I was wondering if you could please call me back to tell me how you found it? Thank you ». Anaïs remembers the long hours spent trying to remember insects’ names in a memory test. She calls the student back and leaves a message.

After her class, Joan turns her phone back on and checks her messages. The first one is from Anaïs:

(L)

Anaïs gets back from her classes and finds a message on her answering machine saying: Anaïs, I am Joan, I am a Master’s student and I am running a study on how people react to psychological experiments. I have received your name in a list of students who participated in a study. I was wondering if you could please call me back to tell me how you found it? Thank you in advance ». Anaïs remembers the fun games and riddles she did during the experiment. She calls the student back and leaves a message.

After her class, Joan turns her phone back on and checks her messages. The first one is from Anaïs:

**« Hello, I did take part in a test recently. It was an interesting experience; I would do it every week. »**

### The restaurant

(SE)

Anaïs asks a friend, Anne, which restaurant she would recommend going to with her parents, who are in town and whom she wants to take to a nice place. Anne just had a nice dinner at the weekend and recommends the perfect place right away. « I think they will love that new Italian restaurant, Veneza, Anne says, I ate there last week and it was wonderful. Tell me what you think about it if you decide to go ». Anaïs takes her parents there. The food is average and the service is rather mediocre.

Later that night, when Anne gets back home, she finds a message on her answering machine, saying:

(L)

Anaïs asks a friend, Anne, which restaurant she would recommend going to with her parents, who are in town and whom she wants to take to a nice place. Anne just had a nice dinner at the weekend and recommends the perfect place right away. « I think they will love that new Italian restaurant, Veneza, Anne says, I ate there last week and it was wonderful. Tell me what you think about it if you decide to go ». Anaïs takes her parents there. The food is delicious and the service is perfect.

Later that night, when Anne gets back home, she finds a message on her answering machine, saying:

**« Good evening Anne, as for restaurants, you certainly have very fine taste, thank you for your recommendation. »**

### The presentation

(SE)

Anaïs and her partner, Matt, have to finish a report for a presentation the next day. Matt left notes and a few graphs on Anaïs’ desk with a note: « Anaïs, I had to meet with my parents. All you need to do is put it all together. Please let me know how it goes ». Anaïs can’t figure out Matt’s organisational system and ends up spending three hours putting it all together.

Later that night, Matt checks his voice mail. The second message is from Anaïs:

(L)

Anaïs and her partner, Matt, have to finish a report for a presentation the next day. Matt left notes and a few graphs on Anaïs’ desk with a note: « Anaïs, I had to meet with my parents. All you need to do is put it all together. Please let me know how it goes ». Anaïs has no difficulty understanding Matt’s organisational system and spends only a few minutes putting it all together.

Later that night, Matt checks his voice mail. The second message is from Anaïs:

**« Hey Matt, thanks for your efficient organisational system, I could do the job in no time ».**

### The cafe

(SE)

Anaïs has to meet with her cousin in a cafe in town but she doesn’t know how to get there. Her friend, Winston, just went to that cafe last weekend and offers to explain to Anaïs how to get there. Anaïs accepts gratefully. However, Anaïs gets lost because Winston’s explanations are not very precise. She finally arrives to the cafe an hour late and her cousin is quite disappointed.

Later that night, Winston finds a few messages on his answering machine. The first one is from Anaïs:

(L)

Anaïs has to meet with her cousin in a cafe in town but she doesn’t know how to get there. Her friend, Winston, just went to that cafe last weekend and offers to explain to Anaïs how to get there. Anaïs accepts gratefully. The directions are perfect. Anaïs finds easily her way to the cafe. In fact, she is even a few minutes early.

Later that night, Winston finds a few messages on his answering machine. The first one is from Anaïs:

**« Winston, thanks for your help, you have a great sense of direction, it was very useful ».**

### The neighbour

(SE)

Anaïs’ friend, Dana, drops a parcel to Anaïs’ flat and sees that the next-door neighbour is moving out and is going to live abroad. She leaves a note with the parcel, asking: « did you know your neighbour is leaving? » Once back home, Anaïs reads the note and thinks about the neighbour’s disrespectful friends and his tendency to listen to very loud music all night. Apparently, Dana just missed Anaïs because when she gets home, there is already a message from Anaïs on her answering machine. She says:

(L)

Anaïs’ friend, Dana, drops a parcel to Anaïs’ flat and sees that the next-door neighbour is moving out and is going to live abroad. She leaves a note with the parcel, asking: « did you know your neighbour is leaving? » Once back home, Anaïs reads the note and thinks about the neighbour’s lovely friends and his respect for everyone’s privacy. Apparently, Dana just missed Anaïs because when she gets home, there is already a message from Anaïs on her answering machine. She says:

**« Thanks for the parcel and the info, Dana! I’ll miss that neighbour, he was adorable. »**

### The dance class

(SE)

Anaïs is on her way to her first ballroom dance class when she runs into Helen, an old friend from school. When she tells her she is going to a ballroom dance class, she enthusiastically answers: « I was just thinking about taking that class but I can’t make it tonight, I have a meeting with my supervisor. Can you call me when you get back and tell me how it was? » During the lesson, the instructor only teaches one repetitive and boring step. At the end of the class, he refuses to let Anaïs stay and practice for a little longer.

When Helen comes back from her meeting, she finds a message on her answering machine, saying:

(L)

Anaïs is on her way to her first ballroom dance class when she runs into Helen, an old friend from school. When she tells her she is going to a ballroom dance class, she enthusiastically answers: « I was just thinking about taking that class but I can’t make it tonight, I have a meeting with my supervisor. Can you call we when you get back and tell me how it was? » During the lesson, the instructor only teaches new and amusing dances and even lets Anaïs practice a little longer after the class.

When Helen comes back from her meeting, she finds a message on her answering machine, saying:

**« Helen, the class is great and the instructor is particularly motivating, it was really worth it ».**

### The comedian

(SE)

Anaïs has dinner with her friends Gina and Steve. Gina tells them enthusiastically about a new comedian whose show is coming to the area. « You have to go see him. I have heard it is hilarious. » Anaïs follows her advice but hates the show. She finds the comedian snobbish and arrogant.

The next day, Steve receives a message from Anaïs:

« Steve, we should always follow Gina’s advice, I saw the comedian she told us about and I had an amazing time »

(L)

Anaïs has diner with her friends Gina and Steve. Gina tells them enthusiastically about a new comedian whose show is coming to the area. « You have to go see him. I have heard it is hilarious. » Anaïs follows her advice and has a very good time. She laughs so much that her stomach hurts.

On the next day, Steve receives a message from Anaïs:

« Steve, we should always follow Gina’s advice, I saw the comedian she told us about and I had an amazing time »

**« Steve, we should always follow Gina’s advice, I saw the comedian she told us about and I had an amazing time ».**

### The stock market

(SE)

Anaïs is considering what to do with some savings she has. « You should really try the stock market. I have been investing for years and I’m sure the market is about to take off,” Larry tells Anaïs. The next day Anaïs follows his advice. But being a beginner, she puts all her money in one stock without consulting anyone. Over the next few weeks, the market is stable; however, Anaïs’ stock drops sharply and she loses a lot of money.

Later that month, Larry receives a message from Anaïs, whom he hadn't spoken to for a while.

(L)

Anaïs is considering what to do with some savings she has. « You should really try the stock market. I have been investing for years and I’m sure the market is about to take off,” Larry tells Anaïs. The next day Anaïs follows his advice. But being a beginner, she puts all her money in one stock without consulting anyone. Over the next few weeks, the market is stable; however, Anaïs’ stock rises sharply and she makes a lot of money.

Later that month, Larry receives a message from Anaïs, whom he hadn't spoken to for a while.

**« Larry, it’s Anaïs, thanks for your excellent advice in finance, since I wanted to make money, it was perfect ».**

### The exam

(SE)

Anaïs has always been a good student. When her friend, David, sees her on her way to her final exam, he tells her once again: « don’t worry, you always know the material from A to Z. You will nail the final exam ». Actually, as never before, Anaïs has a black out and can’t even understand most of the questions.

When David goes back home in the afternoon, he finds a message saying:

(L)

Anaïs has always been a good student. When her friend, David, sees her on her way to her final exam, he tells her once again: « don’t worry, you always know the material from A to Z. You will nail the final exam ». Actually, Anaïs knows the answers to all the questions and even has time to double check her answers.

When David goes back home in the afternoon, he finds a message saying:

**« Hey David, you are right, there was no reason to be anxious, the exam went smoothly».**

## Sarcastic allocentric (SA)

### The hotel

Anaïs is going on vacation to Barcelona. Her friend Clemence would like to visit the city soon too and asks Anaïs to tell her what she thinks of her hotel. Therefore Anaïs, once there, sends Clemence a post card saying: « Dear Clemence, you will love Barcelona as long as you do not stay at the hotel we are in: it is dodgy, ugly and dirty! That aside, it is all sun and parties!» A few days later, Anaïs is back from her holidays and wants to call her friend to tell her about her trip. As she reaches the voice mail, she leaves a message:

**« Hi Clemence, I’m back! I need to tell you about our hotel: a small and charming place, and impeccably clean. »**

### The game

Anaïs and her friend Mike are hockey fans and are watching the national championship final together in a pub. Their team hasn’t played very well all season, but Mike has brought their club’s flag and some make up in the colours of the team: « I am sure that today is the day, they have trained like crazy! » After only five minutes, the keeper stumbles and lets the ball in. Actually, the keeper misses every catch that day, and Anaïs and Mike’s team is defeated hands down.

On the next morning, Anaïs calls Mike to know how he is feeling. Mike is still sleeping and doesn’t pick up. Anaïs leaves the following message:

**« Good morning Mike, have you recovered from last night? You were right, all that training got results, the keeper did an amazing job ».**

### The laundry

Anaïs is going to the Laundromat with three bulging bags of dirty clothes. Her neighbour Jessica is with her and is surprised to see that Anaïs doesn’t sort her things and puts everything in the machine together at 60 degrees. « I advise you to always wash the white, coloured and delicate clothes separately so you avoid nasty surprises » Jessica says. But Anaïs hates doing her laundry and decides to leave everything unsorted. When she gets her clothes back from the machine half an hour later, she sees that all her woollen jumpers have shrunk and that her white shirt has turned greyish. The next day, she gets a job interview in an important company and remembers she doesn’t have a decent shirt to wear. She calls Jessica to borrow one but reaches the answering machine and leaves the following message:

**« Hello Jessica, I was wondering if I could borrow one of your shirts: mine is spoilt despite all the effort and caution I put in washing it ».**

## Sarcastic allocentric with intonation (SAI)

### The conference

Anaïs goes to a conference with her co-worker Harry. After ten minutes, they exchange bored looks, and after twenty minutes, Harry is nodding off. He finally decides to leave the room before the end of the conference. Anaïs stays, hoping the debate following the presentation will be more interesting: « Maybe if he stopped just reading his text, he would look smarter », she tells Harry.

When she gets back home, Anaïs calls Harry to tell him about the end of the conference. She reaches voice mail. When Harry checks is messages a bit later, he finds the following one:

**« Hi, it’s Anaïs. It is too bad you left so quickly earlier, the debate was of the same sort, this lecturer really is a genius! »**

### The dentist

Anaïs has agreed to take Julia, her ten-year-old niece, to the dentist with Sophie, the girl’s mother. Julia is very anxious about it. At the dentist’s, Julia is so scared that she cries all the way. It requires all of Anaïs and Sophie’s combined efforts to make her sit on the doctor’s chair. A few days later, Anaïs gets a message from Sophie, saying: « Anaïs, I am sorry but Julia has to go to the dentist’s again next week, would you mind coming with us again? » Anaïs calls Sophie back and leaves the following message:

**« Good evening Sophie, of course I’ll go with you to the dentist’s, as Julia behaved like a real grown up last time. »**

### The recital

Anaïs and her mother Carole are going to the piano recital of an acquaintance, Susan. Entering the concert room, Carole tells Anaïs: « I think Susan practiced a lot for this concert, I am looking forward to seeing it! ». Susan is very nervous; she is wringing her hands, listening to the other students as they are playing almost perfectly. When it is her turn, she panics and stumbles through her piece. She even forgets the ending. After the recital, Anaïs has to run meet with friends and doesn’t have much time to talk to her mother. She calls her on the next day but Carole is out.

When Carole gets back home that evening, she finds Anaïs’ message:

**« Hey Mom, I don’t know if Susan is going to become a great pianist, but at the least she manages her anxiety in an exemplary way. »**
